# Event-triggered self-regulating integrated PID extreme optimization and fault-tolerant strategy for floating offshore platforms driven by TBG-EDF dual finite-time learning mechanism

**DOI:** 10.1371/journal.pone.0337290

**Published:** 2025-11-24

**Authors:** Chengliang Chao, Yang Liu, Zongkai Wang

**Affiliations:** 1 Xiamen Ocean Vocational College, Xiamen, Fujian, China; 2 School of Navigation and Shipping Shandong Jiaotong University, Weihai, China; 3 Quanzhou Normal University, Quanzhou, Fujian, China; Tongji University, CHINA

## Abstract

Floating offshore platforms (FOPs) face the challenge of anchor chain failures due to their unique operating environments. This directly impacts the platform’s safety and stability. Traditional control methods are often ineffective in addressing anchor chain failures. Therefore, this study proposes a novel event-triggered, self-tuning integrated PID control strategy based on finite-time learning to improve the stability and fault handling capabilities of FOP systems. This study introduces a preset finite-time convergence function based on a time-based generator (TBG) in the control design, combined with a nonlinear error-driven mechanism to achieve finite-time convergence. By integrating a composite variable construction method with neural network approximation techniques, a state mapping mechanism adaptable to mixed uncertainties is established. Furthermore, this study designs a control system that integrates an event-triggered dynamic mechanism with a finite-time convergence paradigm. Through strategic parameter scheduling, this control strategy achieves coordinated optimal configuration and extreme performance optimization under multiple performance metrics in an offline phase. A rigorous stability analysis of the designed control strategy using Lyapunov stability theory demonstrates its effectiveness in terms of asymptotic stability and finite-time convergence. Simulations are conducted on a real FOP system. Results demonstrate that the control strategy effectively addresses anchor chain failures and internal and external uncertain dynamics.

## Introduction

As land resources approach their development limits, the human development vision is gradually extending from land to sea. The vast resource potential of the ocean is becoming a new focus of global strategic layout and sustainable development [1–3]. The ocean contains vast oil and gas wealth, but to access it, you need a stable carrier that can navigate the uncertain marine environment. In this context, floating offshore platforms (FOPs) have gradually entered the field of vision of researchers. Due to its advantages of low investment, strong adaptability, and ease of maintenance, its control system is widely deployed on offshore platforms and barges [4,5]. In this system, the health status of the anchor chain is an implicit variable that cannot be ignored. Because it plays a decisive role in the stability of the entire platform. Once the anchor chain fails, its impact cannot be underestimated. At the very least, it will lead to the anchor position to shift, and at the worst, it will cause the overall instability of the platform structure. The control system may drop in efficiency or even cause serious safety accidents. This series of consequences is enough poses a systemic risk to the entire offshore operations system. Therefore, the occurrence mechanism of anchor chain failure must be systematically revealed, and an effective response plan is also urgently needed. This is not only a practical need at the engineering level, but also the logical starting point for ensuring the safe development of marine energy.

The motion control of an FOP is not a simple mechanical regulation problem, but a complex nonlinear system with constraints, multiple disturbances, and strong coupling. Existing research has revealed the limitations of traditional control methods. They can still give satisfactory responses in static models, but in real marine environments, the control strategy changes at any time. In response to this challenge, researchers have proposed various control strategies. Adaptive sliding mode control and feedback linearization methods have been successfully applied to floating wind turbine control [6,7]. This method achieves independence from the disturbance boundary assumption by self-adjusting the sliding mode gain and completes position or propeller speed convergence within a finite time. High-order sliding mode control and super-torsion algorithm overcome the chattering problem of traditional SISO sliding mode and are effectively used in dynamic positioning systems to ensure control continuity and high robustness [8]. Furthermore, for strong nonlinear coupling scenarios, variable gain high-order sliding mode strategies are used to achieve adaptive gain control and suppress the influence of high-frequency disturbances on platform pitch [9]. At the same time, the robust adaptive scheme of disturbance observer (DOB) combined with dynamic surface control has been proven to be highly effective in nonlinear systems [10,11]. On the one hand, DOB provides real-time disturbance reconstruction; on the other hand, DSC avoids the problems of differential explosion and input saturation, allowing the system to maintain smooth convergence under complex constraints. These methods are not isolated from each other, but complement each other on different technical paths. Sliding mode and its high-order variants provide strong robustness and fast convergence; feedback linearization and dynamic surface architecture solve the complexity of order and modeling dependence; DOB inserts disturbance compensation logic to achieve active response to complex external disturbances [12]; the introduction of adaptive laws ensures that the system no longer relies on “prior parameters”. Especially in the process of moving towards “intelligent control”, such as embedding reinforcement learning or deep neural networks into the control structure, the system can be given the ability to understand environmental changes online and self-optimize control strategies.

In practice, the attitude and control of offshore platforms must seek orderly operation in the face of continuous disturbances, parameter uncertainties, system faults, and resource constraints. Traditional asymptotic stability theory often promises an “ultimate stillness” in the infinite future, but the real world never allows for endless waiting. By constructing Lyapunov functions with singularities or non-smoothness, the system state can converge to the target state in a strictly finite time, which is independent of the initial value, demonstrating remarkable time autonomy [13–15]. In the dynamic control of offshore platforms, the finite time control (FTC) method gives the platform the ability to “quickly recover”, especially in the context of strong sudden disturbances and random waves, its advantages far exceed those of traditional PID [16,17] and LQR [18,19] methods. However, the cost of this speed is the high dependence on model information and the complexity of the nonlinear controller structure. This has triggered extensive exploration of adaptive mechanisms and intelligent approximate methods. Event-triggered control (ETC) is essentially about determining the boundary between “necessity” and “minimum intervention.” Under this method, the system no longer samples at equal intervals, but updates the control only when certain error conditions are met or when a state transition occurs [20,21]. This method dramatically reduces communication and energy consumption. On marine platforms, this mechanism not only alleviates the problem of limited bandwidth for remote control signals but also naturally incorporates a certain fault-tolerant buffer. However, “how to select the trigger threshold” and “how to avoid Zeno behavior” are still unsolved problems in the theoretical community [22,23]. Existing methods mainly rely on a hybrid mechanism of time triggering/event triggering, or introduce a trigger dead zone; however, their practicality requires further verification [24]. The fault-tolerant control problem arises from the fact that the above two types of mechanisms are inevitably challenged in real-world engineering applications. Marine platform control systems often operate under “non-ideal” conditions such as incomplete information, actuator degradation, and sensor drift [25,26]. If the control system cannot perceive and judge the fault memory, the entire platform will go from certainty to out of control. Two primary classifications exist for fault-tolerant control systems: the passive type and the active type. The former is like a shield of redundant structure, and the latter is like a spear of intelligent perception. Active fault tolerance is particularly dependent on fault diagnosis observers and control law reconstruction mechanisms [27]. In recent years, deep learning and reinforcement learning algorithms have been gradually integrated into the marine field to build a closed-loop system architecture with “self-adaptation, self-healing, and self-judgment.” Some advanced studies even attempt to combine the “fast convergence” of finite-time control with the “robust reconstruction” of fault-tolerant systems to construct a “finite-time fault-tolerant controller,” thereby achieving instantaneous recovery of the system under disturbance.

For the fault diagnosis problem, the reference [28] integrates the hybrid model and signal analysis fault diagnosis architecture, cleverly combining the advantages of model prediction and signal analysis, improving the accuracy of fault diagnosis and increasing the response speed. However, this method may still encounter the challenge of performance degradation when facing time-varying faults. To solve this problem, the reference [29] has achieved excellent results in processing complex multi-dimensional sensor data by introducing deep neural networks and convolutional block attention modules. However, how to improve its real-time and generalization capabilities is still a question worth considering in depth. Similarly, the reference [30] provides a new perspective for adaptability and online control optimization in complex dynamic environments based on a data-driven model predictive control method. The reference [31] can effectively cope with complex environmental changes by enhancing the deep learning radial basis function network. However, the huge computational complexity of the deep learning model and its need for real-time updates restrict its application in real-time control. On this basis, the dual multi-vector model predictive control method solves the coupling and constraint problems in multi-input and multi-output systems [32]. Nevertheless, in the face of the uncertainty of system parameters, there is still a need to further improve robustness. As mentioned above, damping technology plays a vital role in improving the dynamic stability of the platform. However, in complex marine environments, there is still room for optimization of these technologies. Reference [33] cleverly balances economic efficiency and system reliability by adopting a reliability-based design optimization method. However, these optimization methods face high computational complexity and real-time challenges in practical applications. With the rise of digital twin technology, FOPs monitoring has ushered in new opportunities. Reference [34] proposed a digital twin condition monitoring method. By monitoring the system status in real time, the fault warning capability is significantly enhanced. However, this method’s high dependence on computing resources is still a bottleneck for its widespread application. For fault compensation, active fault-tolerant control methods based on fuzzy logic systems are an important direction to meet the challenge. Through the clever combination of fuzzy control and adaptive technology, these methods can adjust the dynamic uncertainty, actuator faults and sensor faults of the system in real time. More importantly, this method can effectively handle the nonlinear characteristics of the system without relying on an accurate mathematical model. By adjusting the control strategy online, it ensures that the system can still operate stably when a fault occurs. The optimal active fuzzy fault-tolerant control scheme proposed in reference [35] can cope with various types of actuator and sensor faults. By combining fuzzy logic systems with backstepping technology, the stability and robustness of the system under complex fault conditions are ensured. In addition, for unknown multivariable nonlinear systems, reference [36] proposed an adaptive fuzzy fault-tolerant tracking control method. This method cleverly uses fuzzy control to approximate system faults and overcomes the problems of external disturbances and unknown control signs through robust control. Similarly, the active fuzzy fault-tolerant control scheme proposed in reference [37] has shown good effectiveness under multiple faults and interferences and can effectively handle different types of sensor and actuator faults. In addition, the general active fault-tolerant control scheme in reference [38] provides a more general solution. By combining fuzzy logic systems with backstepping techniques, a variety of fault types can be handled, including additive and multiplicative faults, actuator nonlinearities, and external disturbances. These research results provide strong technical support for fault tolerance and stability control of floating platforms.

Inspired by previous work, this paper explores a fascinating and fundamental question: Is there a clever control strategy that enables designers to clearly determine the convergence time and control accuracy of the FOP control system in advance under the event-triggered mechanism? More importantly, can this control strategy simultaneously cope with the complex situations that FOP must face in the real world, such as dynamic uncertainty, unknown external disturbances, and even sudden failures of anchor chains? To answer this question, this paper proposes an innovative control strategy. The main contributions of this paper are as follows:

1. For the first time, the problem of anchor chain faults is incorporated into the control scenario of FOP, and two types of loss-of-effectiveness (LOE) faults and bias faults are considered. Compared with [4,5], the scheme developed in this paper compensates for the fault factor to ensure that the system can still maintain good performance when a fault occurs.
2. This paper develops a fast convergence scheme driven by a dual finite time learning mechanism. Compared with the finite time control scheme in [3,11,14], the finite time control scheme designed in this paper can quickly adjust the controller parameters through the dual effects of time drive and error feedback, thereby achieving faster convergence speed.
3. This paper develops a self-tuning integrated PID mechanism with event-triggered input. Compared with the traditional PID control scheme, the integrated PID mechanism designed in this paper not only has a concise structure, but also solves the unstable characteristics of the traditional PID control scheme when facing nonlinear dynamics.

FOP control is evolving towards a fusion of intelligence and adaptability. While traditional PID and swarm tuning methods are robust, they struggle to cope with transient sea conditions and the uncertainties of faults. Research on FOP control has evolved along three main lines: first, event-triggered mechanisms to manage communication and energy consumption; second, finite time and learning laws to pursue rapid and stable convergence; and third, fault tolerance and extreme value optimization to empower the system with self-healing and self-optimization capabilities. The proposed TBG-EDF dual finite time learning-driven, event-triggered, self-tuning integrated PID framework embodies the intersection of these three elements. It uses learning to promote perception, triggering to control the pacing, and extreme value-driven optimization to enable the system to stabilize under disturbances, self-repair under faults, and maintain rational order within the complex and ever-changing ocean dynamics. This approach can be viewed as a learning-enhanced, event-triggered, fault-tolerant hybrid control, harmoniously integrating traditional control concepts with intelligent evolutionary mechanisms.

## 1. Problem formulation and preliminaries

**Fig 1:**
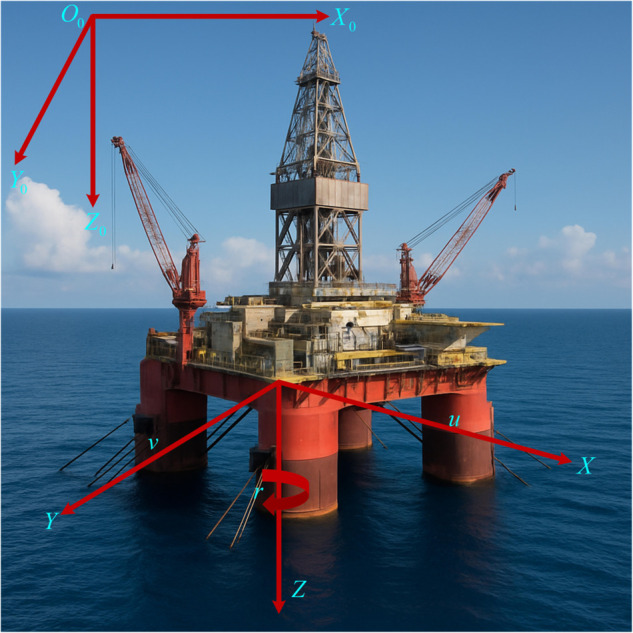
Coordinate system.

In order to clearly describe the dynamic behavior of the floating platform, we must first define two right-handed coordinate systems that are related to each other in the observer’s field of view, as shown in Fig 1. The first coordinate system is the Earth-fixed coordinate system *O*_*o*_, *X*_*o*_, *Y*_*o*_, *Z*_*o*_. We arbitrarily choose a point on the surface of the earth as its origin *O*_*o*_, and then carefully point its axes to the north, east, and the center of the earth. The second coordinate system is called the body-fixed coordinate system *OXYZ*. Its origin *O* falls at the core of the center of gravity of the platform, and its axes are respectively facing the rear of the platform, the starboard side, and the sky. Therefore, any posture and movement of the platform can be accurately and elegantly presented in this coordinate system that moves with it. We know that the movement of the platform is slow and low-speed, so we are more concerned with its dynamic characteristics on the sea surface under the gentle caress of the waves. Based on this understanding, we further established a low-frequency nonlinear mathematical model to describe the three key motions of FOP: pitch, roll and yaw [39]:

$$\left\{ \begin{array}{c} \dot{\varsigma }=\kappa \left(\alpha \right)\omega \\ \chi \dot{\omega }+D\omega =\rho +\delta (t) \end{array}\right.$$ (1)

In the Earth-fixed coordinate system, the position of the platform is represented by vector $\varsigma ={\left[x,y,\alpha \right]}^{\text{T}}$, where *x* and *y* represent the longitudinal and lateral positions of the platform in the plane, respectively, and *α* represents its yaw angle. Correspondingly, the velocity state of the platform is described by vector $\omega ={\left[u,v,r\right]}^{\text{T}}$, where *u* and *v* are the longitudinal and lateral velocity components of the platform in its own coordinate system, respectively, and *r* is the rate of change of the yaw angle. The conversion between coordinate systems depends on the rotation matrix κ(α), which is in the following form:

$$\kappa \left(\alpha \right)=\left[\begin{array}{ccc} \cos\alpha & -\sin\alpha & 0\\ \sin\alpha & \cos\alpha & 0\\ 0 & 0 & 1 \end{array}\right]$$ (2)

Matrix χ represents the total inertia of the system including the added mass effect, while *D* is the equivalent damping matrix, which takes into account the energy dissipation of the mooring cable on the system in the longitudinal, transverse and yaw directions. Together, they determine the dynamic response of the platform under disturbance. However, the FOP control system cannot physically provide infinite restoring force. The tension of the anchor cable is limited by its material properties and structural design. In order to ensure the stable operation of the system and the safety of the anchor cable structure, the mooring force on the platform must be physically constrained. This constraint can be expressed in the form of Eq (3). Its essence is to impose a saturation limit on the change of the anchor cable tension, thereby suppressing nonlinear drastic changes and preventing it from exceeding the design safety boundary. At the same time,

$$\rho_{i}=\{\begin{array}{ccc} \rho_{i\;\max}, & if & \rho_{ci}>\rho_{i\;\max}\\ \rho_{ci}, & if & \rho_{i\;\min}<\rho_{ci}<\rho_{i\;\max}\\ \rho_{i\;\min}, & if & \;\rho_{ci}<\rho_{i\;\min} \end{array}i=1,2,3$$ (3)

where i=1,2,3 correspond to the dynamic response of the platform in the three degrees of freedom of surge, sway and yaw. The expected input calculated by the controller in each degree of freedom is denoted as $\rho_{ci}$. Its function is to offset disturbances and stabilize the attitude; while the maximum and minimum resultant forces or torques that the FOP control system can provide in this direction constitute a finite boundary, limiting the physical range of the actual control action. The control error is defined as the difference between the actual control signal and the expected control command, and its magnitude reflects the degree of deviation between the system’s execution capability and the ideal state. The external environmental disturbance is expressed in vector form, $\delta (t)={\left[\delta_{u}(t),\delta_{v}(t),\delta_{r}(t)\right]}^{\text{T}}$, representing the surge disturbance force, sway disturbance force and yaw disturbance torque acting on the platform respectively. It represents the inevitable dynamic disturbance source of the system in open waters.

The fault of the anchor chain is caused by many factors. Waves and ocean currents continue to exert pressure, and the chain links fatigue and fission under repeated loads; salt water corrosion silently weakens the strength of the metal; the seabed topography restricts and kinks the anchor chain, generating abnormal stress. Material defects and poor welding are like microscopic hidden dangers, which are infinitely magnified in dynamic stress. If the automatic control system fails to adjust the platform’s attitude properly, the anchor chain may be pulled to the limit in an instant. We cannot ignore the deep logic behind these physics and engineering. The relationship between the faults suffered by the platform and the control input can be described as follows:

$$\tau_{i}^{f}=j_{i}\left(t\right)\varrho_{i}+c_{i}\left(t\right),\;i=u,v,r$$ (4)

where $0<j_{i}\left(t\right)<1$ represents the LOE faults, and $c_{i}\left(t\right)>0$ represents the bias faults suffered by the system.

**Assumption 1:** The parameter matrix *D* and the external perturbation δ(t) are unknown and bounded.

**Assumption 2:** The motion trajectory signal $\varsigma^{d}$ of FOP is smooth and differentiable.

**Remark 1:** When the FOP motion deviates, the root of the problem is often not the external environment such as wind and waves, but the subtle cracks inside the system. LOE faultsare caused by the decline of the power system: damage to the propeller blades, aging of the motor, loose gears, unstable power supply - these physical defects make the input signal discounted and difficult to execute truthfully. The root cause of the bias faults lurks between the sensor and the signal path: the gyroscope offsets the zero point due to temperature drift, the ADC sampling voltage fluctuates, electromagnetic interference is mixed with false signals, and even the parameters are set incorrectly during system initialization.

**Control objective:** This study aims to construct an event-triggered self-adjusting integrated PID limit optimization and fault-tolerant strategy for FOP driven by a TBG-EDF dual finite-time learning mechanism, so that the FOP control system can accurately guide the position (x,y) and heading angle of the FOP to any small neighborhood of the desired target $\varsigma^{d}=\left[x^{d},y^{d},\alpha^{d}\right]$ within the finite time *a* preset by the user under given assumptions. The core of the control strategy is that, on the one hand, time *a* is a controllable, system-independent offline parameter, which reflects the initiative of the method; on the other hand, all signals of the entire closed-loop system are always bounded during operation, ensuring the unity of convergence and system stability. This design method combines the advantages of finite-time learning and feedback mechanisms, and realizes the extreme optimization of “controllability” and control accuracy in a nonlinear dynamic environment.

## 2. TBG-based predefined FTC function

The nonlinear and time-dependent nature of TBG requires that its initial and final values comply with certain constraint conditions. Only when these conditions are met can the function be defined as TBG. TBG is a mathematical structure that is subject to boundary and time evolution laws. If (*a*) satisfies the following properties, then the function can be called TBG, denoted by (*a*)

(1) [(a)]=1. (*a*), $\left(\dot{a}\right)$, $\left(\ddot{a}\right)$ exhibits continuous behavior and is confined within a bounded range.

(2) (a)=1 for ∀a>b with *b* being the predefined setting time.

(3) Within the interval ∀a∈[0,b], both the first and second derivatives of (*a*) exist and exhibit strictly decreasing behavior.

To further reveal the specific connotation of this principle, let us examine the following representative examples [40]:

$$\left(a\right)=\left\{ \begin{array}{cc} b^{-\hbar }{\left(b-a\right)}^{\hbar } & \left(\hbar \geq 2\right)a<b\\ 0 & a>b \end{array}\right.$$ (5)

$$\left(a\right)=\left\{ \begin{array}{cc} b^{-\hbar }{\left(b-a\right)}^{\hbar }e^{-a} & \left(\hbar \geq 2\right)a<b\\ 0 & a>b \end{array}\right.$$ (6)

$$\left(a\right)=\left\{ \begin{array}{cc} b^{-\hbar }{\left(b-a\right)}^{\hbar }\lambda \left(a\right) & \left(\hbar \geq 2\right)a<b\\ 0 & a>b \end{array}\right.$$ (7)

where λ(a) evolves over time, maintaining continuity without an increasing trend, showing a monotonically decreasing or constant time dependence. And λ(0)=1. λ(a) is second-order differentiable. In addition, the function itself and its first- and second-order derivatives are all within a bounded range, reflecting an inherent stability and controllability.

Based on the inherent properties of TBG (*a*), we further construct a class of finite-time functions with predefined behaviors, which are as follows:

$$\partial \left(a\right)={\left[\left(1-\rho \right)\left(a\right)+\rho \right]}^{-1}$$ (8)

where ρ∈(0,1) is design parameter.

## 3. Main results

**Fig 2:**
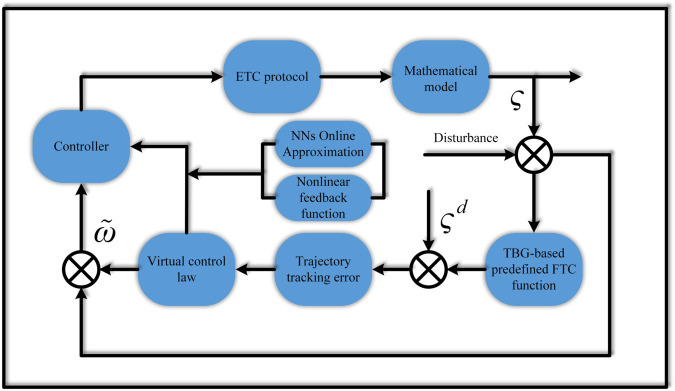
Schematic diagram of the control scheme design process.

The research framework of this paper is shown in Fig 2. This section will design the event-triggered self-regulating integrated PID control law in two steps. Before formally constructing the control law, we first introduce two error variables as the logical starting point for subsequent derivation:

$$\tilde{\varsigma }=\varsigma -\varsigma^{d}$$ (9)

$$\tilde{\omega }=\omega -\hat{\omega }$$ (10)

where $\varsigma^{d}$ is the reference value of the platform motion trajectory. $\hat{\omega }$ is the filtered form of the platform reference velocity $\omega^{d}$ through the following Levant differentiator:

$$\left\{ \begin{array}{c} {\dot{\varphi }}_{1}=\beta_{1}\\ \beta_{1}=-\varphi_{1}{\left|\varphi_{1}-\omega^{d}\right|}^{\frac{1}{2}}sgn\left(\varphi_{1}-\omega^{d}\right)+\beta_{2}\\ {\dot{\beta }}_{2}=-\sigma_{2}sgn\left(\varphi_{2}-\pi \right) \end{array}\right.$$ (11)

where $\omega^{d}$ is the input. $\sigma_{1}$, $\sigma_{2}$ are positive definite design parameters.

**Step 1:** Take the time derivative of the error variable and use Eqs (1), (9) and (10) to obtain:

$$\dot{\tilde{\varsigma }}=\dot{\varsigma }-{\dot{\varsigma }}^{d}=\kappa \left(\alpha \right)\left(\tilde{\omega }+\hat{\omega }\right)-{\dot{\varsigma }}^{d}$$ (12)

The dummy control variable $\omega^{d}$ is designed as

$$\omega^{d}=\kappa^{\text{T}}\left(\alpha \right)\left[-\gamma_{11}\xi \left(\varsigma \right)+\varsigma^{d}\right]$$ (13)

where $\xi \left(\varsigma \right)=\varsigma +\gamma_{12}\varsigma ({\left\|\varsigma \right\|}^{2}+{\vartheta_{\varsigma }}^{2}{)}^{-1}$ is the nonlinear error driving function about ς. $\gamma_{11}>0$ and $\gamma_{12}>0$ are design parameters.

**Step 2:** According to Assumption 1 and Eq (10), we can get:

$$\chi \dot{\tilde{\omega }}j\left(t\right)\rho +c\left(t\right)+\delta (t)-D\omega -\chi \dot{\hat{\omega }}$$ (14)

where Dω is unknown. This paper uses neural network technology to reconstruct it, as follows:

$$-D\omega =\zeta^{T}\pi \left(\upsilon \right)+\mu$$ (15)

where $\zeta^{T}$ is the neural network weight. π(υ) is the neural network function. *u* is the approximation error. Substituting Eq (15) into Eq (14) yields:


$$\chi \dot{\tilde{\omega }}=\chi \dot{\omega }-\chi \dot{\hat{\omega }}$$

$$\leq j\left(t\right)\rho +\left\|c\left(t\right)+\delta (t)+\mu \right\|+\left\|\zeta^{T}\right\|\left\|\pi \left(\upsilon \right)\right\|+\left\|-\chi \dot{\hat{\omega }}\right\|$$

≤j(t)ρ+Ξo (16)

where $\Xi =\max\left\{\left\|c\left(t\right)+\delta (t)+\mu \right\|,\left\|\zeta^{T}\right\|,1\right\}$, o=‖π(υ)‖+2.

The relative threshold event triggering mechanism is defined as follows:

$$\left\{ \begin{array}{c} \rho \left(t\right)=P\left(t_{k}\right),\forall t\in \left[t_{k},t_{k+1}\right)\\ t_{k+1}=\inf\left\{t\in R|\left|\tilde{P}\left(t\right)\right|\geq \phi \rho \left(t\right)+\eta \right\} \end{array}\right.$$ (17)

where *ϕ* and *η* are positive definite design parameters. $\tilde{P}\left(t\right)=P\left(t\right)-\rho \left(t\right)$ is the measurement error of the system.

According to Eq (17), we can get:

$$P\left(t_{k}\right)=\left[1+\kappa_{1}\left(t\right)\phi \right]\begin{array}{c} {}*20l\rho \end{array}\left(t\right)+\kappa_{2}\left(t\right)\eta$$ (18)

where $\lambda_{1}\left(t\right)$ and $\lambda_{2}\left(t\right)$ are time-varying parameters, which satisfy $\left|\lambda_{1}\left(t\right)\right|\leq 1$, $\left|\lambda_{2}\left(t\right)\right|\leq 1$. Further, we can get:

$$\begin{array}{c} {}*20l\rho \end{array}\left(t\right)=\frac{P\left(t\right)}{1+\lambda_{1}\left(t\right)\phi }-\frac{\kappa_{2}\left(t\right)\eta }{1+\lambda_{1}\left(t\right)\phi }$$ (19)

Combined with Eq (14), the error dynamics equation of the system can be rewritten as:

$$\chi \dot{\tilde{\omega }}\leq \frac{j\left(t\right)}{1+\lambda_{1}\left(t\right)\phi }P\left(t\right)-\frac{j\left(t\right)}{1+\lambda_{1}\left(t\right)\phi }\lambda_{2}\left(t\right)\eta +c\left(t\right)+\Xi o$$ (20)

Construct the integration variable as follows:

$$\Phi =\varpi \left[m\left(-\varpi \lambda_{2}\left(t\right)\eta +c\left(t\right)\right)\right]\;=\varpi \varphi$$ (21)

where $\varpi =\frac{j\left(t\right)}{1+\kappa_{1}\left(t\right)\phi }=m^{-1}$ is unknown bounded nonlinear function greater than 0.

The error integration variables are constructed as follows:

$$\chi C=\chi \tilde{\omega }+\varepsilon_{1}\chi \int \tilde{\omega }dt+\varepsilon_{2}\chi \dot{\tilde{\omega }}$$ (22)

where $\varepsilon_{1}>0$ and $\varepsilon_{2}>0$ are design parameters.

Taking the time derivative of Eq (22) yields

$$\chi \dot{C}=\chi \dot{\tilde{\omega }}+\varepsilon_{1}\chi \tilde{\omega }+\varepsilon_{2}\chi \ddot{\tilde{\omega }}$$ (23)

Substituting Eqs (18) and (19) into Eq (23), we can obtain

$$\chi \dot{C}\leq \varpi P\left(t\right)+\varpi \tilde{O}+\varpi \hat{O}$$ (24)

where $\hat{O}$ is the estimate of O. $\tilde{O}=O-\hat{O}$, $O=\max\left\{m\Xi ,m\varepsilon_{1}\chi ,m\varepsilon_{2}\chi \right\}$.

Based on the above analysis, the control law designed as

$$P\left(t\right)=\kappa_{P}\tilde{\omega }+\kappa_{I}\int \tilde{\omega }dt+\kappa_{D}\dot{\tilde{\omega }}$$ (25)

where $\kappa_{P}=k_{P}+\hat{k}$, $\kappa_{I}=k_{I}+\hat{k}$, $\kappa_{D}=k_{D}+\hat{k}$, $k_{P}=-\gamma_{21}[1+\gamma_{22}{\left({\left\|C\right\|}^{2}+{\vartheta_{C}}^{2}\right)}^{-1}]$, $k_{I}=-\gamma_{21}\varepsilon_{1}[1+\gamma_{22}{\left({\left\|C\right\|}^{2}+{\vartheta_{C}}^{2}\right)}^{-1}]$, $k_{D}=-\gamma_{21}\varepsilon_{2}[1+\gamma_{22}{\left({\left\|C\right\|}^{2}+{\vartheta_{C}}^{2}\right)}^{-1}]$, $\hat{k}=\hat{O}$, $\dot{\hat{O}}=\psi_{1}({\left\|C\right\|}^{2}-\psi_{2}\hat{O})$, with $\psi_{1}>0$, $\psi_{2}>0$.

The Lyapunov function is selected for the closed-loop control system as follows:

$$Q_{V}=\frac{1}{2}{\tilde{\varsigma }}^{2}+\frac{1}{2}\chi C^{2}+\frac{1}{2\psi_{1}}{\tilde{O}}^{2}$$ (26)

Taking the time derivative of Eq (26) and substituting Eqs (12) and (24) into it, we can obtain

$${\dot{Q}}_{V}\leq \tilde{\varsigma }\left[\kappa \left(\alpha \right)\left(\tilde{\omega }+\hat{\omega }\right)-{\dot{\varsigma }}^{d}\right]+\varpi CP\left(t\right)\;\;+C\varpi \tilde{O}+C\varpi \hat{O}-\tilde{O}\left({\left\|C\right\|}^{2}-\psi_{2}\hat{O}\right)$$ (27)

Substituting Eqs (13) and (25) into Eq (27), we can obtain

$${\dot{Q}}_{V}\leq -\gamma_{11}\xi \left(\varsigma \right)\tilde{\varsigma }+\kappa \left(\alpha \right)\tilde{\varsigma }\tilde{\omega }-\varpi C\gamma_{21}\xi \left(C\right)\;\;+C\varpi \tilde{O}-{\left\|C\right\|}^{2}\tilde{O}+\psi_{2}\tilde{O}\hat{O}$$ (28)

According to the description in reference [3], Eq (28) can be further simplified as:

$${\dot{Q}}_{V}\leq -\gamma_{11}{\tilde{\varsigma }}^{2}+\gamma_{11}\gamma_{12}\vartheta_{C}-\gamma_{11}\gamma_{12}\left\|\tilde{\varsigma }\right\|\;\;-\gamma_{21}C^{2}+\gamma_{21}\gamma_{22}\bar{\varpi }\vartheta_{C}-\gamma_{21}\gamma_{22}\left\|C\right\|\;\;+\frac{\psi_{2}}{2}{\left\|O\right\|}^{2}-\frac{\psi_{2}}{4}\tilde{O}-\frac{\psi_{2}}{4}{\left\|\tilde{O}\right\|}^{2}\;\leq -\zeta^{o}Q_{V}-\zeta^{t}{Q_{V}}^{\frac{1}{2}}+\zeta_{Q_{V}}$$ (29)

where $\zeta^{o}=\min\left\{2\gamma_{11},2\gamma_{21},\frac{\psi_{2}}{2}\right\}$, $\zeta^{t}=\sqrt{2}\min\left\{\gamma_{11}\gamma_{12},\gamma_{21}\gamma_{22},\frac{\psi_{2}}{4}\right\}$, $\zeta_{Q_{V}}=\gamma_{11}\gamma_{12}\vartheta_{C}+\gamma_{21}\gamma_{22}\bar{\varpi }\vartheta_{C}-\frac{\psi_{2}}{4}\tilde{O}$.

According to Eq (29), we can get:

$${\dot{Q}}_{V}\leq -\tau \zeta^{o}Q_{V}-\left(1-\tau \right)\zeta^{o}Q_{V}-\zeta^{t}{Q_{V}}^{\frac{1}{2}}+\zeta_{Q_{V}}$$ (30)

If $Q_{V}>\frac{\zeta_{Q_{V}}}{\tau \zeta^{o}}$, then we can get

$${\dot{Q}}_{V}\leq -\left(1-\tau \right)\zeta^{o}Q_{V}-\zeta^{t}{Q_{V}}^{\frac{1}{2}}$$ (31)

In addition, according to the lemma in [10], $Q_{V}$ can be stabilized in the residual set $\Omega_{Q_{V}}=\left\{Q_{V}:Q_{V}\leq \frac{\zeta_{Q_{V}}}{\tau \zeta^{o}}\right\}$ in a finite time, and the stability time is:

$$T\leq \frac{4}{\left(1-\tau \right)\zeta^{o}}\ln\left[\frac{\left(1-\tau \right)\zeta^{o}\sqrt{Q_{V}}\left(0\right)+\zeta^{t}}{\zeta^{t}}\right]$$ (32)

where $Q_{V}\left(0\right)$ is the initial value of $Q_{V}$.

Furthermore, according to Eq (26), we can get $\frac{1}{2}{\tilde{\varsigma }}^{\text{T}}\tilde{\varsigma }\leq Q_{V}\leq \frac{2\zeta_{Q_{V}}}{\tau \zeta^{o}}$, that is,

$$\Omega_{Q_{V}}=\left\{{\tilde{\varsigma }}^{\text{T}}\in R|\left\|{\tilde{\varsigma }}^{\text{T}}\right\|\leq \sqrt{\frac{2\zeta_{Q_{V}}}{\tau \zeta^{o}}}\right\}$$ (33)

According to Eq (29), we can get ${\dot{℘}}_{v}\leq -\zeta^{o}J_{V}$  +  $\zeta_{Q_{V}}$, which means that ${℘}_{v}$ s bounded. Based on the boundedness of ${℘}_{v}$ and Eq (26), it can be determined that $\tilde{\varsigma }$, C and $\tilde{O}$ are bounded. Since C is bounded, $\tilde{\omega }$, $\int \tilde{\omega }dt$ and $\dot{\tilde{\omega }}$ are bounded. Based on the boundedness of $\tilde{\omega }$ and C, it can be concluded that $\omega^{d}$ and *ω* are bounded. Then it can be determined that the control law P(t). This guarantees that all signals within the closed-loop tracking framework are uniformly bounded.

By taking the derivative of the measurement error $\tilde{P}\left(t\right)$, we can obtain:

$$\frac{d\left[\tilde{P}\left(t\right)\right]}{dt}=\frac{d}{dt}{\left[\tilde{P}\left(t\right)*\tilde{P}\left(t\right)\right]}^{\frac{1}{2}}=sgn\left[\tilde{P}\left(t\right)\right]\dot{\tilde{P}}\left(t\right)\leq \left|\dot{P}\left(t\right)\right|$$ (34)

From Eq (25), we can get:

$$P\left(t\right)=-\gamma_{12}C-\gamma_{12}\gamma_{22}\dot{C}+\gamma_{12}\gamma_{22}C{\left({\left\|C\right\|}^{2}+{\vartheta_{C}}^{2}\right)}^{-2}+C\dot{\hat{O}}$$ (35)

Since all signals in a closed-loop tracking system are bounded, there exists a constant ℓ that satisfies $\left|\dot{P}\left(t\right)\right|\leq \ell$. When the measurement error $\tilde{P}\left(t\right)=0$, we can get $\underset{t\to t_{k+1}}{\lim}\tilde{P}\left(t\right)=\phi \left|\rho \left(t\right)\right|+\eta$. Therefore, there must be a time interval t^*^ that satisfies $t^{*}\geq \frac{\phi \left|\rho \left(t\right)\right|+\eta }{\ell }$. Therefore, the Zeno phenomenon will not occur.

**Remark 1:** This strategy combines a dual finite-time learning mechanism, event-triggered regulation, and integrated PID limit optimization. Consequently, its control law involves multiple layers of nested operations and real-time learning updates. In practical platform control systems, real-time requirements are typically in the millisecond range, and complex learning laws and extreme value optimization algorithms can lead to computational delays. Future research is exploring “lightweight learning laws” based on model reduction or online approximation, or employing parallel hardware acceleration to improve real-time performance.

**Remark 2:** This paper utilizes a dual finite-time learning mechanism, TBG-EDF, to enable the system to achieve self-awareness and self-correction within a limited timeframe. Event-triggered, self-tuning PID integrated control ensures the platform only acts when truly needed, maintaining optimal performance with minimal energy consumption. Extreme value optimization and fault-tolerance strategies empower the system to maintain balance and stability amidst uncertainty and failures. Potential applications span floating wind power, deep-sea drilling, dynamic positioning, and ballast compensation. The system can maintain steady state amidst the chaos of wind, waves, and loads, and rapidly recover from sudden failures and disturbances.

## 4. Simulation studies

In order to verify the actual effect of the control law proposed in this paper, we applied it to the model of the eight-point moored semi-submersible FOP “Kan Tan 3” and carried out numerical simulation. This platform is 91 meters long, with a beam span of 71 meters, a designed operating depth of 250 meters, and a displacement of 25,240 tons. In order to facilitate simulation analysis, we scaled it down. The inertial mass matrix and hydrodynamic parameter matrix can be found in [41]. The controller parameters are selected as $\sigma_{1}=0.1$, $\sigma_{2}=10$, $\gamma_{11}=0.1$, $\gamma_{12}=0.5$, ϕ=0.2, η=0.03, $\gamma_{21}=0.3$, $\gamma_{22}=0.015$, $\psi_{1}=0.3$, $\psi_{2}=0.02$. Furthermore, we utilize the Integrated Absolute Error (IAE) and Mean Integrated Absolute Control (MIAC) metrics to evaluate steady-state performance and energy usage. The results of this evaluation are shown in Tables 1 and 2.

**Table 1:** Comparison of the control performance of IAE.

$\text{IAE}=\frac{1}{t_{f}}\int_{0}^{t_{f}}\left|P(t)\right|dt,\;i=u,v,r$			
	**TBG-EDF-ETFTC scheme**	**TBG-EDF-FTC scheme**	**The scheme in [3]**
${\tilde{\varsigma }}_{x}$	10.52	10.52	11.27
${\tilde{\varsigma }}_{y}$	9.31	9.25	10.35
${\tilde{\varsigma }}_{\psi }$	9.15	9.03	9.82

**Table 2:** Comparison of the control performance of MIAC.

$\text{MIAC}=\frac{1}{t_{f}}\int_{0}^{t_{f}}\left|P(t)\right|dt,\;i=u,v,r$			
	**TBG-EDF-ETFTC scheme**	**TBG-EDF-FTC scheme**	**The scheme in [3]**
$P_{u}$	1.85	1.78	1.65
$P_{v}$	1.52	1.46	1.32
$P_{r}$	0.73	0.68	0.51

In order to construct a more physically realistic external environment, this section introduces the disturbance model proposed in the [28]. This model is based on the NORSOK wind spectrum and the JONSWAP wave spectrum, and simulates typical sea breeze and wind-driven wave conditions. The results of numerical tests conducted under this sea condition show that the proposed method still maintains good robustness under complex sea conditions, which is sufficient to cope with the uncertainty and nonlinear characteristics of external disturbances.

This peper focuses on two types of LOE faults and bias faults, and their mathematical forms are as follows:

$$\left\{ \begin{array}{c} j_{u}\left(t\right)=0.5+0.5\exp\left(-0.1t\right)\\ j_{v}\left(t\right)=0.3+0.7\exp\left(-0.2t\right)\\ j_{r}\left(t\right)=0.6+0.4\exp\left(-0.15t\right) \end{array}\right.$$ (36)

$$\left\{ \begin{array}{c} c_{u}\left(t\right)=0.2+0.5\cos\left(0.2t\right)\\ c_{v}\left(t\right)=0.3+0.4\sin\left(0.1t\right)\\ c_{r}\left(t\right)=0.5+0.2\cos\left(0.2t\right) \end{array}\right.$$ (37)

**Fig 3:**
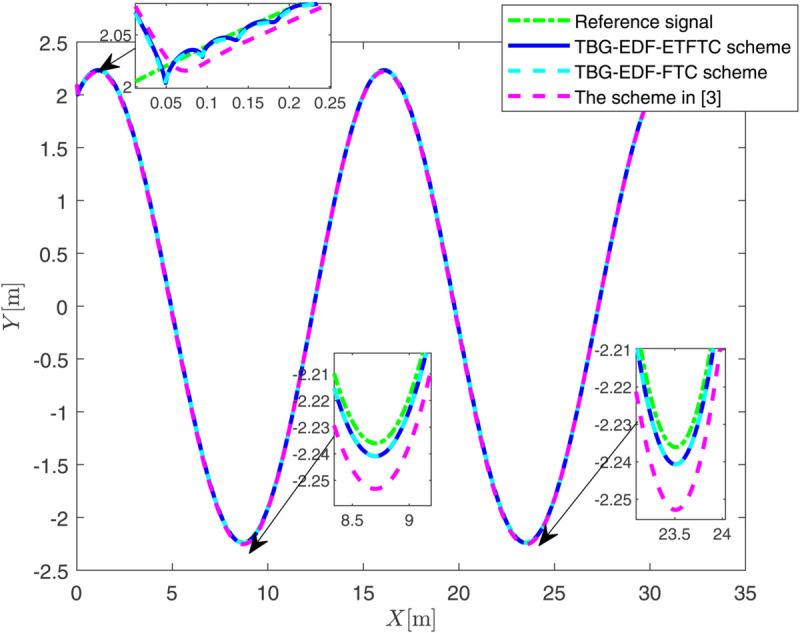
Motion trajectory of FOP under three control schemes.

**Fig 4:**
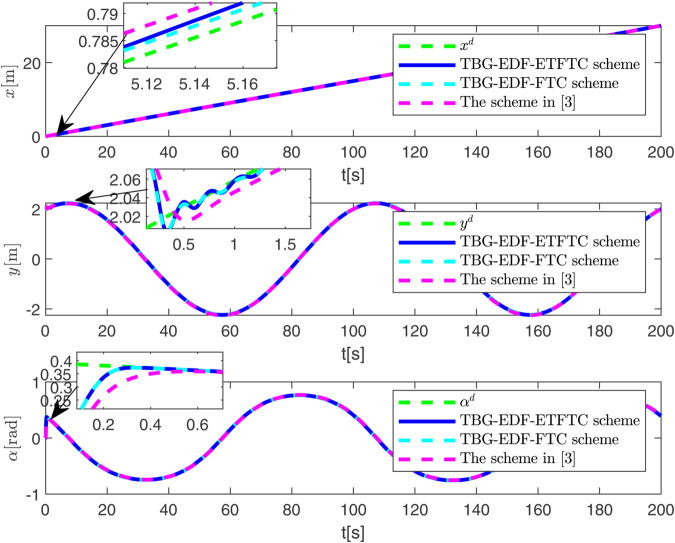
Tracking effect of FOP on reference signal under three control schemes.

**Fig 5:**
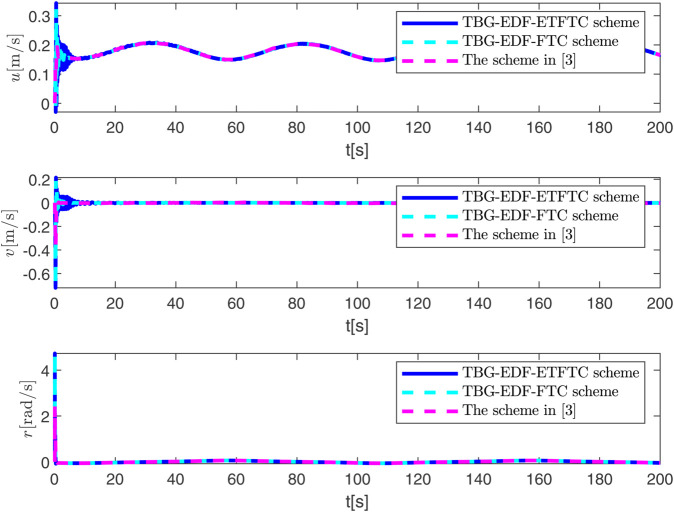
Changes in FOP velocity under three control schemes.

**Fig 6:**
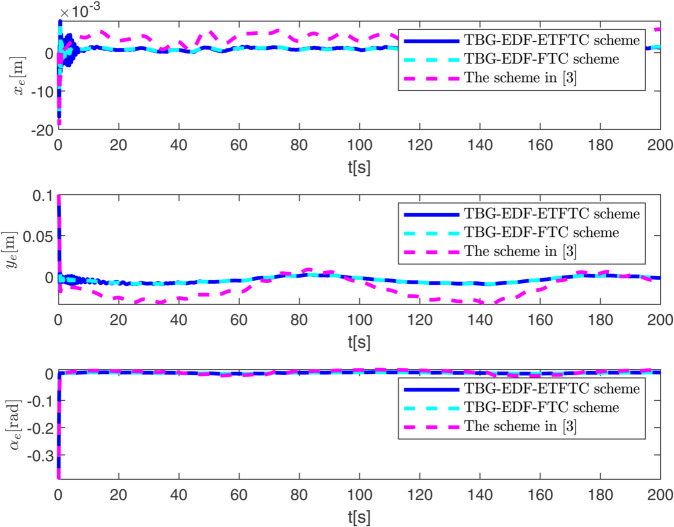
Evolution of tracking error over time.

**Fig 7:**
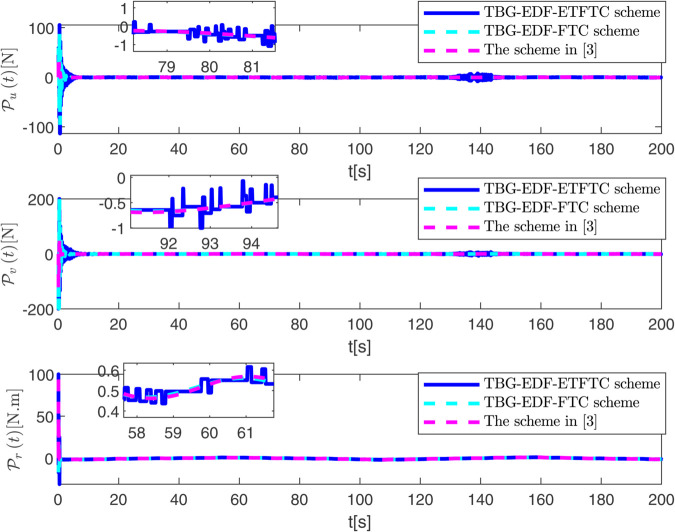
Evolution of control input over time.

**Fig 8:**
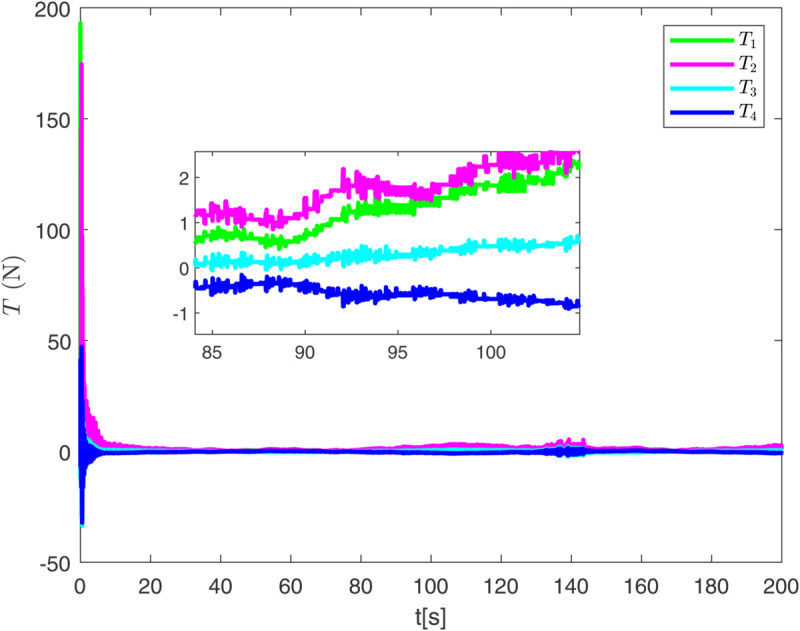
Evolution of anchor chain tension over time under event-triggered control scheme.

**Fig 9:**
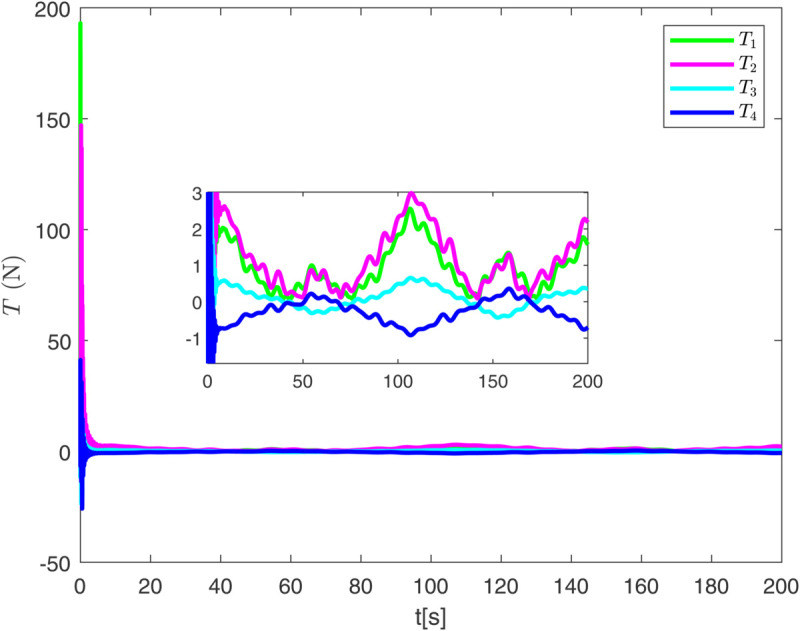
Evolution of anchor chain tension over time under finite time control scheme.

**Fig 10:**
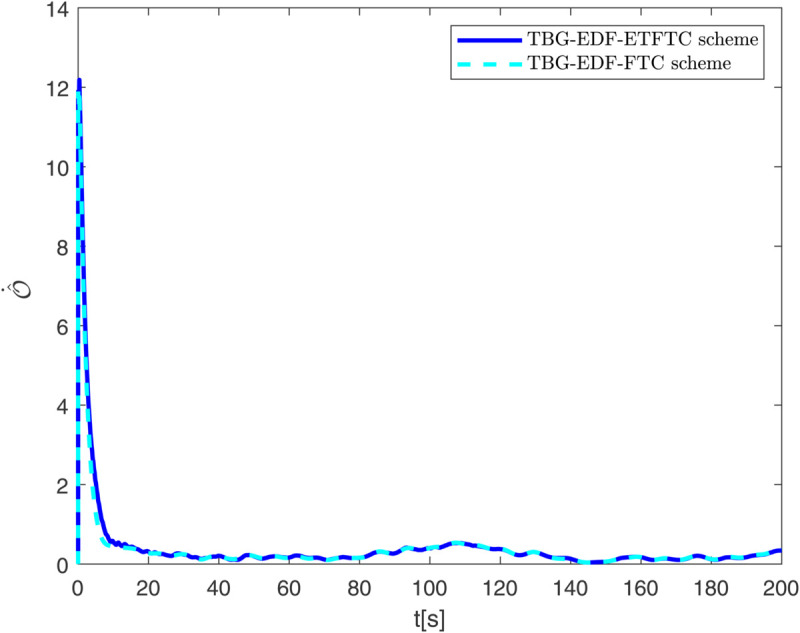
Evolution of the estimated value $\hat{O}$ over time.

**Fig 11:**
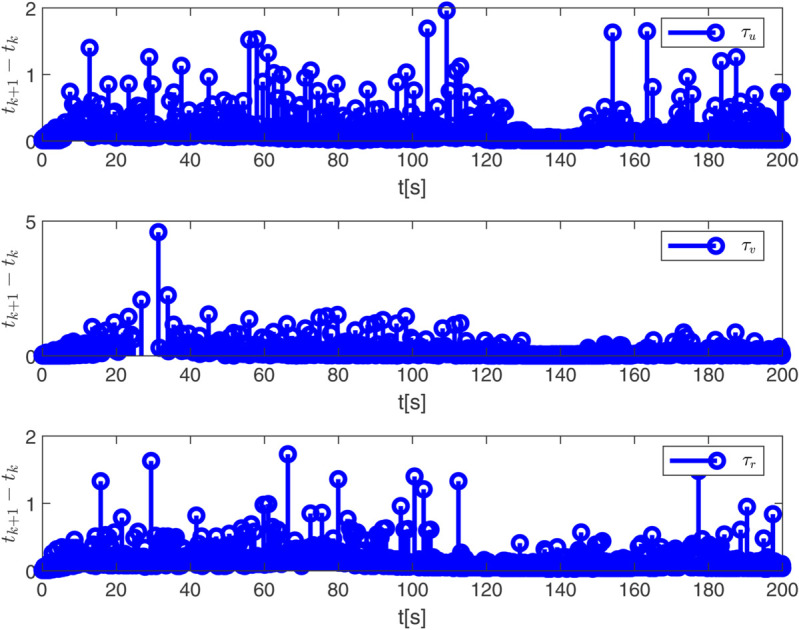
Event triggering effect.

By observing Figs 3 to 11 and Tables 1 to 2, at the moment when the anchor chain fault suddenly appeared (see Figs 3 and 4), the two proposed control schemes did not “barely maintain” but returned to the reference trajectory with a shorter adjustment time and smaller overshoot. The smooth transition is not accidental. It comes from the time domain shaping of the transition process by TBG and the instant estimation of the unknown term by EDF, which enables the closed loop to maintain bounded gain and robustness margin when the actuator constraints and structural mismatch coexist. As a result, the speed and attitude do not drift, but enter a long-term constant attraction domain (Fig 5). This means that even if the external disturbance is applied again, the system will only fluctuate within a small range and will not become unstable. In terms of accuracy (Fig 6), TBG-EDF-FTC gives the best steady-state error; when we introduce event triggering to obtain TBG-EDF-ETFTC, the accuracy is slightly compromised but almost negligible, and both are significantly better than the literature [3]. This is repeatedly confirmed by the indicators such as IAE/ITAE in Tables 1 and 2: we use fewer updates and communications to buy almost the same tracking quality. More importantly, the trajectories of the control input and anchor chain tension (Figs 7–9) exhibit no high-frequency chattering or secondary shocks. The evolution within the saturation boundary demonstrates that the control law remains viable, and the fault does not induce coupled oscillations. Composite uncertainty estimation (Fig 10) demonstrates that the EDF provides rapid and bounded compensation for the combined disturbances of environmental loads, time-varying parameters, and unmodeled dynamics, thereby preventing steady-state bias accumulation and eliminating tail oscillations. Under the influence of event triggering, the system controller updates only 2231, 2296, and 1686 times (Fig 11), significantly reducing computational and communication overhead while maintaining nearly equivalent error levels. In summary, under the dual constraints of anchor chain failure and input saturation, the proposed control scheme achieves maximum order with minimal “action,” mitigating the degradation caused by the fault while approaching the performance limits of the FOP system under limited resource constraints.

## 5. Conclusion

Aiming at the problem of anchor chain faults in FOP during operation, this paper proposes an event-triggered self-adjusting integrated PID control strategy based on finite time learning. This strategy comprehensively introduces the preset finite time convergence function of TBG and the nonlinear error driving mechanism, realizes the rapid and stable control of the system in a finite time, and enhances the system’s response to disturbances and uncertainties. By integrating the composite variable construction method and the neural network approximation technology, this paper establishes a state mapping mechanism for mixed uncertainty, thereby realizing effective modeling and compensation control of system bias failures, external disturbances and dynamic nonlinear uncertainties. At the same time, with the help of the collaborative design of the event trigger mechanism and the finite time convergence paradigm, parameter optimization and extreme performance configuration under multi-dimensional performance indicators are realized in the offline stage of the controller, which improves the overall control efficiency and resource utilization of the system. This paper provides an effective technical path for solving the anchor chain failure control problem in the FOP system, and provides a theoretical basis and technical reference for subsequent related research. In the future, we will consider incorporating extreme sea conditions and the coupled platform-mooring-riser-propulsion dynamics into a unified model, evaluating the finite-time convergence properties and stability margins under model mismatch and strong nonlinearities. Furthermore, in the control design, we will consider cyberattacks in networked control and design security event triggering mechanisms and control laws with detection and fault tolerance capabilities.
